# *Candida* Surveillance in Surgical Intensive Care Unit (SICU) in a Tertiary Institution

**DOI:** 10.1186/s12879-015-0997-6

**Published:** 2015-07-03

**Authors:** Yi Xin Liew, Jocelyn Teo, Irene Ai-Ling Too, Cecilia Cheng-Lai Ngan, Ai Ling Tan, Maciej Piotr Chlebicki, Andrea Lay-Hoon Kwa, Winnie Lee

**Affiliations:** Department of Pharmacy, Singapore General Hospital, Block 8 Level 2, Outram Road, Singapore, 169608 Singapore; Division of Nursing, Department of SICU, Singapore General Hospital, Outram Road, Singapore, 169608 Singapore; Department of Pathology, Singapore General Hospital, Outram Road, Singapore, 169608 Singapore; Department of Infectious Diseases, Singapore General Hospital, Outram Road, Singapore, 169608 Singapore; Emerging Infectious Diseases, Duke-National University of Singapore Graduate Medical School, 8 College Rd, Singapore, 169857 Singapore; Department of Pharmacy, Faculty of Science, National University of Singapore, Block S4A, Level 3, 18 Science Drive 4, Singapore, 117543 Singapore

**Keywords:** Beta-D-glucan, *Candida* surveillance, Colonization, Surgical intensive care unit

## Abstract

**Background:**

Colonization of patients occurs before development into invasive candidiasis. There is a need to determine the incidences of *Candida* colonization and infection in SICU patients, and evaluate the usefulness of beta-D-glucan (BDG) assay in diagnosing invasive candidiasis when patients are colonized.

**Methods:**

Clinical data and fungal surveillance cultures in 28 patients were recorded from November 2010, and January to February 2011. Susceptibilities of *Candida* isolates to fluconazole, voriconazole, amphotericin B, micafungin, caspofungin and anidulafungin were tested *via* Etest. The utilities of BDG, *Candida* score and colonization index for candidiasis diagnosis were compared *via* ROC.

**Results:**

30 BDG assays were performed in 28 patients. Four assay cases had concurrent colonization and infection; 23 had concurrent colonization and no infection; three had no concurrent colonization and infection. Of 136 surveillance swabs, 52 (38.24 %) were positive for *Candida spp*, with *C. albicans* being the commonest. Azole resistance was detected in *C. albicans* (7 %). C. glabrata and C. tropicalis were, respectively, 100 and 7 % SDD to fluconazole*.* All 3 tests showed high sensitivity of 75–100 % but poor specificity ranging 15.38–38.46 %. BDG performed the best (AUC of 0.89).

**Conclusions:**

Despite that positive BDG is common in surgical patients with *Candida spp* colonization, BDG performed the best when compared to CI and CS.

## Background

Invasive candidiasis (IC) in critically ill patients is associated with high mortality and morbidity [1, 2]. While early diagnosis and treatment of IC will improve survival rates significantly, clinical and radiological signs are non-specific and develop late in the course of the disease. Furthermore, the epidemiology of *Candida* infections have evolved, with an increased proportion of non-*albicans* due to frequent use of azoles [3]. This in turn affects the efficacy of the empiric antifungal regimen used in these high risk patients who are critically ill. Therefore, it is important to understand the epidemiology and incidence of *Candida* colonization and infections in a surgical intensive care unit setting, where the patients are at higher risks for IC, in order for effective treatment to be instituted early [4].

Rendering prompt antifungal therapy is also limited by current culture-based diagnostic techniques for the diagnosis of *Candida* bloodstream infection which lack sensitivity, with up to 50 % of cases remaining undiagnosed. Moreover, these tests have long lag-time, ranging from 85 to 124 h [5]. Hence, early detection of a component of fungal cell wall, beta-d-glucan (BDG), may be a useful diagnostic marker for fungal infections. In a recent study comparing colonized patients to those with IC, BDG was superior to blood cultures in identifying deep-seated candidiasis. The combination of blood cultures and BDG had a sensitivity of 79 %, suggesting that BDG may be a useful adjunct to blood cultures, to aid in the identification of some patients who are currently undiagnosed [6]. Our study aims to determine the incidence and type of *Candida* colonization and infection in SICU, as well as to relate the observed BDG concentrations to patients’ fungal status.

## Methods

### Screening

The study was carried out in a tertiary care hospital in Singapore over a three-month period (November 2010 and January to February 2011). Patients above 18 years of age, who were admitted to Singapore General Hospital (SGH) surgical intensive care unit (SICU) for at least 48 h, were prospectively identified from Electronic Health Records (Eclipsys Sunrise, Allscripts Corp., Chicago, IL). The following patients were excluded from the study: aged younger than 18; neutropenic (defined as absolute neutrophil count <0.5 × 10^9^/L); pregnant/lactating; or were colonized/had documented or possible fungal infections as diagnosed by an infectious diseases specialist (including fungal infections other than *Candida spp.*) prior to SICU admission .

This study was approved by the SingHealth Centralized Institutional Review Board (IRB no. 2010/494/D) and waiver of consent was granted.

### Surveillance cultures

Surveillance cultures for *Candida spp.* were obtained from eligible patients, with the first samples taken on the third day post ICU admission, and weekly thereafter until discharge from the ICU or death. This corresponded to the days which BDG levels were determined. The following sites were cultured for fungal microorganisms: i) rectum (*i.e.* rectal swabs or feces), ii) urine (if any), iii) respiratory tract (*e.g.* bronchoalveolar lavage specimens), iv) wound (if any), and v) blood. Results were considered positive in the presence of *Candida* growth in the culture medium. The different *Candida* isolates were further speciated at the microbiology laboratory.

All included patients would be eventually classified into three groups with respect to their fungal status: 1) neither colonized nor infected, 2) *Candida* species colonization without IC, and 3) *Candida* species colonization with proven IC.

Colonization was considered unifocal when *Candida* species were isolated from one site and considered multifocal when *Candida* species were simultaneously isolated from various noncontiguous foci, even if two different *Candida* species were isolated.

### Diagnosis of *Candida* infections

Proven IC will require one of the following criteria: 1) Histologically confirmed candidiasis from a specimen obtained by needle aspiration or biopsy from a normally sterile site (other than mucous membranes); or 2) Isolation of *Candida species* in a sample obtained by a sterile procedure (including a freshly placed [<24 h ago] drain) from a normally sterile site in a patient with consistent clinical manifestations; or 3) documentation of one or more blood culture(s) that yielded a *Candida* species in a patient with consistent clinical manifestations [7].

Candidal peritonitis is defined as the isolation of *Candida* species in a peritoneal sample obtained by laparotomy, including perforation of an abdominal organ, dehiscence of an intestinal suture with peritonitis, severe acute pancreatitis, or presence of peritoneal catheter for dialysis. Catheter-related candidemia will be considered in patients who have an intravascular device and one or more positive cultures of blood samples obtained from the peripheral vein, clinical manifestations of infection (*e.g.*, fever, chills, and/or hypotension), and no apparent source for bloodstream infection (with the exception of the catheter), as well as a positive catheter culture. Candiduria is defined as the presence of at least 10^4^ colony-forming units/mL of the same *Candida* species.

The decision to treat a patient with antifungal drugs during the course of this study was entirely dependent on the attending physician’s clinical judgment. For all study patients on antifungal agents, details of therapy as well as clinical and microbiological information were collected.

### BDG assay

Serum samples were collected from all eligible patients on (i) the third day of ICU admission and (ii) once a week there after until ICU discharge, death or occurrence of IC.

Blood samples (15 mL) were collected in three tubes without anticoagulant, centrifuged at 1800 rpm for 10 min, separated into aliquots, and stored at −80 °C until analysis. Detection of BDG was performed using Fungitell assays (Associates of Cape Cod Incorporated, MA, US) in duplicates by the serology and immunology laboratory in our institution according to the manufacturer’s instructions. Negative controls (blood from healthy volunteers) were included. Results were regarded as valid if both duplicates were in the same category (both positive and negative) and duplicates did not differ by more than 20 %. Detection of positive BDG is defined as BDG level of ≥ 80 pg/ μL.

### Data collection

The following variables were recorded for each patient: age, gender, date of ICU admission, dates of ICU and hospital discharge, reason for ICU admission, underlying diseases, concomitant infections, number and sites of *Candida* colonization and risk factors (treatment with corticosteroids with a daily dose ≥20 mg prednisolone for at least 2 weeks, use of broad spectrum antibiotics or antimicrobials within 10 days before the study, use of mechanical ventilation before the day of inclusion in the study, and urinary catheter in place on the day of inclusion). Type of surgery (abdominal vs non-abdominal, elective vs urgent) and the number of major procedures performed before and during ICU stay were registered. Acute Physiology and Chronic Health Evaluation (APACHE) II and Charlson comorbidity index were also recorded at ICU admission.

### *Candida* score and Candida colonization index

*Candida* score (CS) and colonization index (CI) were calculated on the days whenever BDG levels were determined. According to Pittet’s definitions, the CI was defined as the ratio of the number of distinct non-blood body sites colonized by *Candida* species to the total number of body sites cultured [8]. Patients with CI ≥ 0.5 were considered heavily colonized. The CS was calculated as follows (variables coded as absent = 0, present = 1): total parenteral nutrition × 1, plus surgery × 1, plus multifocal *Candida* colonization × 1, plus severe sepsis × 2. A CS ≥ 3 accurately selected patients at high risk for IC.

### Anti-fungal susceptibility testing

Minimum inhibitory concentrations (MICs) of amphotericin B, fluconazole, voriconazole, anidulafungin, caspofungin and micafungin were tested for each non-duplicate isolate using the Etest method performed in accordance to manufacturer’s recommendations (bioMérieux, Marcy l’Etoile, France). Briefly, Etests were carried out on plates containing RPMI agar supplemented with 2 % glucose and buffered to pH 7.0 with morpholinepropanesulfonic acid (Sigma-Aldrich, St Louis, MO, US). The inoculum (0.5 McFarland-adjusted cell suspension in 0.85 % NaCl) was swabbed in three directions on the entire RPMI-agar plate, and the E-test strip was applied after excess moisture had been absorbed into the agar. The plates were incubated at 35 °C, and MICs were read after 24 h.

MICs were interpreted in accordance to Clinical and Laboratory Standards Institute (CLSI) species-specific clinical breakpoints, where available [8]. Epidemiological cut-off values were used when breakpoints are not proposed by CLSI.

### Statistical analysis

Categorical variables were expressed as frequencies and percentages, and continuous variables as medians and range. Kruskal Wallis and Chi-square (or Fisher’s exact) tests were used to analyze continuous and categorical variables, respectively. Sensitivity and specificity were calculated for BDG, Candida score and CI. Receiver operator curves (ROC) was used to compare sensitivity and specificity between assays.

## Results

### Study population

**Fig. 1:**
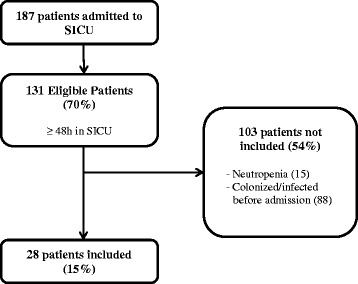
Inclusion of study participants

Out of 187 patients admitted to SICU during the study period, 155 patients were excluded because of lack of fulfillment of the inclusion criteria (Fig. 1). Only 28 patients met the criteria for recruitment. Characteristics of SICU patients are presented in Table 1. They had a median age of 62 (32–85) years old. Upon SICU admission, the median APACHE II score was 20 (5–42) and Charlson Co-morbidity index was 5 (0–16). The median length of ICU stay was 6.5 (3–71) days. Nineteen patients underwent emergency operations; three patients underwent elective operations while six patients did not have any operations. Fourteen patients had abdominal surgery while the remaining 8 patients had non-abdominal related surgeries. There were two patients staying beyond one week in SICU stay and had a second BDG assay done. The APACHE II score, number of risk factors, CI (a second round of surveillance swabs were done) and CS were re-calculated for these two patients, when second sets of BDG assays were performed. Hence, there were 30 cases (with BDG) classified for analysis in this study.

**Table 1:** Characteristics of SICU patients

Colonization Status	No. of cases	Age	Apache II Score	Charlson Comorbidity Score	No of risk factors
Colonized & Infected	−4	69	22	4	4
Colonized & Non-infected					
Unifocal	5	63	16	4	2
Multifocal	18	62	26	6	2
Non-colonized	3	57	16	4	2

There were three (10.0 %) patients who were neither colonized nor infected. Twenty-three (76.7 %) patients were colonized without infection. Of these, five patients were colonized at one site and 18 at two or more sites. Three patients with multi-focal colonization and one patient with unifocal colonization developed IC.

Out of the three patients with multi-focal colonization, two had disseminated Candida *infections* involving multiple sites. Patient one was admitted into SICU for severe sepsis post-brevis flap surgery. A CT abdominal pelvis scan was done and new pancreatic collections were observed. Drainage was performed and the fluid was cultured, isolating *C. tropicalis*. In addition, *C. tropicalis and C. albicans* were also isolated from her blood. Anti-fungal treatment (fluconazole) was started five days later when the blood culture result was available and subsequently treatment was changed to caspofungin. Finally, the patient underwent an exploratory laparotomy with cholecystectomy, pancreatic necrotomy and further drainage of intra-abdominal collections. She demised within a week post-operatively from acute pancreatitis. Patient two, who had underlying adenocarcinoma of the colon, underwent an exploratory laparotomy for a perforated duodenal ulcer. Intraoperative findings included ulcer perforation and gross peritonitis with *C. albicans* isolated from the peritoneal fluid and blood. Immediately post-surgery, the patient deteriorated rapidly, and demised four days after. Anti-fungal therapy was not instituted as the culture and susceptibilities result were not reported in time. The third patient with multi-focal colonization was admitted for septic shock due to a perforated antral ulcer which required an emergency partial gastrectomy. In week one of ICU stay, his peritoneal fluid then grew *Candida* non-*albicans*, for which he was treated with fluconazole upon the availability of culture result. The patient improved and was discharged from SICU. For the patient with unifocal colonization, he was admitted to SICU for septic shock secondary to acute cholangitis. Within 24 h post-ERCP, *C. albicans* was isolated from the bile, obtained *via* a bile drain placed intra-operatively, and his IC episode was subsequently treated with fluconazole when culture result was available. The patient improved and was discharged from SICU.

Five cases received empiric anti-fungal therapy after a new onset of fever while receiving broad-spectrum antibiotics and none of them had culture-proven candidiasis. Two patients underwent intra-abdominal surgeries (exploratory laparotomies) for ischemic bowel resection and intestinal obstruction. One patient had acute necrotizing pancreatitis with cholangiocarcinoma, and another patient had an above knee amputation done, which was complicated with worsening acidosis post-surgery. One patient had intra-abdominal sepsis secondary to abscess which was drained, but received no intra-abdominal surgery. The median (range) time to administration of anti-fungals from onset of sepsis was five days (4–18). Four patients received echinocandins while the remaining one received fluconazole for empiric therapy. These five patients had BDG median readings of 108.33 (46.16–276.23) pg/μL, with median CI of 0.67 (0.33–0.75), and median CS of 3 (3–4). Two of the five patients died (the patient with acute necrotizing pancreatitis and the patient who had an above knee amputation).

### BDG levels

The BDG levels attained by the patients are presented in Table 2. Ten BDG levels were negative with median reading of 45.01 (14.95–68.20) pg/μL while 20 BDG levels were positive at median reading of 189.45 (90.65–>500) pg/μL, including the 4 patients with IC. The median BDG value in group 1, 2 and 3 were 112.14 (90.65–201.47) pg/μL, 104.58(14.95–>500) pg/μL, and 338.72 (124.62–>500) pg/uL respectively. There was no significant statistical difference between the median BDG values in the three groups (*p* = 0.07). Only two patients had serial BDG assay done due to prolonged stay (more than one week) in SICU. On day three of SICU admission, one patient had elevated BDG reading of >500 pg/μL with CI of 1and CS of 4, while the other patient had low BDG reading of 28.84 pg/μL with CI of 0.5 and CS of 5. On day seven of SICU admission, the patient with elevated BDG continued to have elevated BDG reading of >500 pg/μL and CI of 1. Blood culture was now positive, confirming the diagnosis of IC. The CS had increased to five. The patient with low BDG reading continued to have low BDG reading of 43.86 pg/μL with CI of 0.5 and CS of 3.

**Table 2:** BDG levels, CI and CS in different groups

Colonization Status	No. of cases (N)	Median BDG (pg/μl)	No of cases (N)				
			Median BDG Range (pg/ul)			CI ≥ 0.5	CS ≥ 3
			<60	60–80	>80		
Colonized & Infected	4	338.72	0	0	4	3	4
Colonized & Non−infected							
Unifocal	5	108.33	1	0	4	1	5
Multifocal	18	85.58	6	3	9	18	15
Non−colonized	3	112.14			3	0	2

### CI and CS

Eight of the calculated CI was <0.5 while 22 of the calculated CI was ≥0.5. Nineteen patients (82.6 %) had CI ≥0.5 and 4 patients (17.4 %) with CI of <0.5 in group 2. Three patients (75.0 %) in group 3 had CI ≥0.5. There was significant statistical difference between the three groups (*p* = 0.02).

Four CS were <3 while 26 CS were ≥3. Two patients in group 1 had CS ≥3 while 1 patients had CS <3. Twenty patients had CS ≥ 3 while 3 patients had CS <3 in group 2. All patients (100 %) in group 3 had CS of ≥3. There was no significant statistical difference between the three groups (*p* = 0.6).

### Comparison of BDG, CI and CS for diagnosis of IC

**Fig. 2:**
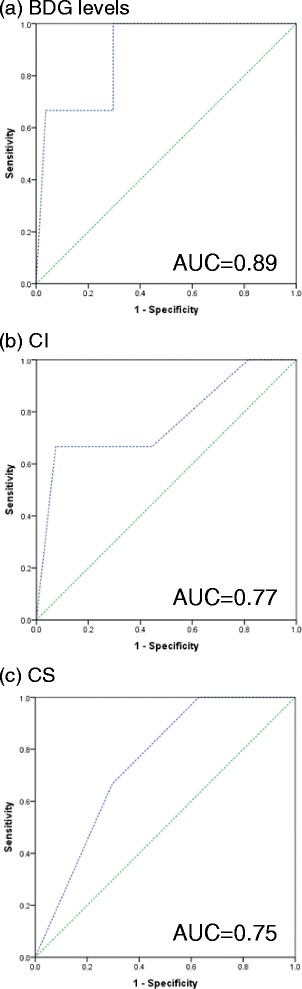
ROC for differentiating IC using (**a**) BDG levels; (**b**) CI; (**c**) CS

Table 3 summarizes the diagnostic accuracy of the BDG values *versus* CI and CS in the diagnosis of IC. All three tests had good sensitivity (75–100 %) but poor specificity. BDG fared the best in terms of specificity at 38.46 % while CS had the poorest specificity at 15.38 %. In view of the low specificity of CS, we recalculated the sensitivity and specificity at a cut-off of 4. This increased the specificity to 53.85 % but sensitivity fell to 50 %. The ROCs of BDG, CI and CS are presented in Fig. 2. BDG performed the best with an AUC of 0.89.

**Table 3:** Accuracy of BDG, CI and CS in diagnosing IC

	Sensitivity (%)	Specificity (%)	PPV (%)	NPV (%)
BDG > 80	100 (40.23–100)	38.46 (20.25–59.42)	20.00 (5.86–43.67)	100 (68.97–100.00)
CI ≥ 0.5	75 (20.34–95.88)	26.92 (11.62–47.79)	13.64 (3.06–34.94)	87.50 (47.38–97.93)
CS ≥ 3	100 (40.23–100)	15.38 (4.45–34.84)	15.38 (4.45–34.89)	100 (40.23–100)
CS ≥ 4	50.00 (8.30–91.70)	53.85 (33.39–73.39)	14.29 (2.20–42.84)	87.50 (61.62–98.08)

### *Candida* surveillance cultures and anti-fungal susceptibility

The results of the surveillance cultures for *Candida spp.* are presented in Table 4. Each positive swab could yield more than one *Candida* isolate. Of 136 swabs cultured from different anatomic sites of 28 SICU patients, 52 (38.2 %) were positive for *Candida spp*. These isolates included *C. albicans* (n = 32), *C. glabrata* (n = 27), *C. dubliniensis* (n = 11), *C. tropicalis* (n = 17) and *C. parasilopsis* (n = 3) (Table 2). Twelve patients (40 %) had more than one type of *Candida* species from the various sites and specimens tested. Overall, the frequency of *Candida* isolation is highest in the rectum (80 %), followed by sputum (74 %), urine (35 %), wound (33 %) and blood (6 %). The antifungal susceptibilities are presented in Table 5. Azole resistance was detected in *C. albicans* (7 %). C. glabrata and C. tropicalis were, respectively, 100 and 7 % SDD to fluconazole*.*

**Table 4:** Species spectrum of *Candida* species isolated from different anatomic sites of SICU patients

Site	*C. albicans*	*C. dubliniensis*	*C. glabrata*	*C. tropicalis*	*C. parasilopsis*
Blood	1	0	2	1	0
Urine	3	0	2	0	0
Rectal	13	6	14	7	2
Sputum	14	5	8	9	1
Wound	1	0	1	0	0
Total	32	11	27	17	4

**Table 5:** Antifungal susceptibilities

Candida species	Anti-fungal	MIC 50	MIC Range	%S	%SDD/I	%R
C. albicans	Fluconazole	0.380	0.064–≥ 512	87	7	7
(n = 15)						
	Voriconazole	0.008	0.008–≥ 64	87	7	7
	Anidulafungin	0.003	≤0.002–0.012	100	0	0
	Caspofungin	0.016	≤0.002–0.064	100	0	0
	Micafungin	0.012	0.002–0.032	100	0	0
	Amphotericin B	0.064	≤0.002–0.250	100	-	0
C. glabrata	Fluconazole	8	0.125–24	-	100	0
(n = 14)						
	Voriconazole	0.125	0.004–0.75	-	-	-
	Anidulafungin	0.032	≤0.002–0.125	100	0	0
	Caspofungin	0.032	≤0.002–0.125	100	0	0
	Micafungin	0.016	0.004–0.032	100	0	0
	Amphotericin B	0.125	≤0.002–0.250	100	-	0
C. tropicalis	Fluconazole	1.5	0.38–4	89	11	0
(n = 9)						
	Voriconazole	0.064	0.016–0.38	67	33	0
	Anidulafungin	0.006	≤0.002–0.094	100	0	0
	Caspofungin	0.032	≤0.002–0.190	100	0	0
	Micafungin	0.023	0.016–0.032	100	0	0
	Amphotericin B	0.190	≤0.002–0.250	100	0	0
C. parapsilosis	Fluconazole	-	0.25–0.5	100	0	0
(n = 2)						
	Voriconazole	-	0.012–0.023	100	0	0
	Anidulafungin	-	0.38–0.75	100	0	0
	Caspofungin	-	0.38–0.75	100	0	0
	Micafungin	-	0.25–0.5	100	0	0
	Amphotericin B	-	0.064–0.5	100	-	0
C. dubliniensis	Fluconazole	0.5	0.125–≥ 512	-	-	-
(n = 8)						
	Voriconazole	0.023	0.004–≥ 64	-	-	-
	Anidulafungin	0.006	≤0.002–0.032	-	-	-
	Caspofungin	0.016	≤0.002–0.094	-	-	-
	Micafungin	0.047	0.012–0.094	-	-	-
	Amphotericin B	0.006	≤0.002–0.25	-	-	-

## Discussion

ICU patients are at higher risk for IC than patients who are in the general ward [9]. Among the known risk factors for IC, *Candida* colonization is probably the most important one as it indicates that patients have an endogenous source of candida [10]. It was noteworthy that all recruited patients in this study were immunocompetent and, 73 % had CI ≥ 0.5 upon first surveillance; with *Candida albicans* as the predominant species isolated in the surveillance cultures. Given that this study is conducted in the SICU, it is not surprising that most of the patients had candida scores ≥ 3. Interestingly, despite the high CI and CS scores indicating the higher risk for IC in these patients, only 4 cases of IC were detected in this study. This suggests that relying on either CI or CS alone may over predict for IC in these patients and lead to unnecessary use of antifungal agents.

In this study, 5 out of 14 patients with intra-abdominal sepsis received empiric antifungal treatment and all were colonized (CI 0.33 to 0.75) with BDG levels ranging from 46 to 276 pg/μl. Empiric antifungals were generally initiated within 7 days of ICU stay, after patients had received a range of wide-spectrum antibiotics and yet did not improved clinically. Unfortunately, only 2 out of the 14 patients with intra-abdominal sepsis survived, underscoring the high case-fatality rates and thus, the need for earlier detection of occult invasive candidiasis and institution of antifungal treatment. One well-known strategy is the pre-emptive approach relying on fungal biomarkers *e.g.* beta-D-glucan.

We evaluated the use of BDG, CS and CI to aid earlier detection of patients with suspected IC. Presently, BDG is widely used as a diagnostic marker for invasive fungal infections and is included as indirect microbiological criteria for the diagnosis of probable IFI in the revised EORTC definitions [7] . This was based on the results from studies evaluating the use of BDG to select patients with candida colonization and through serial readings, to guide preemptive antifungals patients in these high risk patients [11, 12].

Using the FDA approved cut-off of ≥80 pg/ μl, Mohr reported a BDG sensitivity and specificity of 91 and 57 % respectively [13]. A more recent study by Tissot reported sensitivity and specificity of 83 and 52 % in patients with culture-negative intraabdominal sepsis [14]. These results are relatively comparable to those observed in this study where the sensitivity was 100 % and specificity was 40 %. Our lower specificity could be contributed by the high number of false positives in our patient cohort. The median BDG of the 3 non-colonized and non-infected patients was 112.14 pg/ μl (Range 91–125 pg/μL), above the manufacturer’s cutoff value of 80 pg/μl. This anomaly may be explained by the fact all non-colonized patients underwent intra-abdominal surgery which is a surrogate for the use of surgical gauzes; a known cause of falsely elevated BDG [15]. Abdominal surgery involving transection of the gastrointestinal tract also increases the potential for dissemination of BDG in the bloodstream. Additionally, 2 out of 3 of these patients had concurrent bacterial infections, another potential cause of reactivity with the BDG assay [15]. These observations concur with that reported by Tissot *et al.* where 87 and 62 % of non-colonized patients in their study cohort with false-positive BDG had concurrent bacteremia and abdominal surgery respectively [14].

In the SICU, the high proportion of patients who received abdominal surgery may possibly render the use of BDG readings to “rule in” IC inefficient. On the contrary, using the BDG to “rule out” IC may be more applicable given its high negative predictive value observed in this and previous studies. A prospective pilot study on pre-emptive anidulafungin using BDG by Hanson reported similar PPV and NPV of 20.7 and 100 % respectively [11]. As PPV and NPV are influenced by disease prevalence, we believe that this was due to the similar low IC rates observed in our studies. Serial BDG readings > 80 pg/μl had been suggested to increase the specificity of this test without compromising on the NPV [11]. Furthermore, trending of serial BDG levels and subsequent observation of a decreasing gradient was associated with successful echinocandin therapy in one study. Baseline and post-treatment may potentially be useful as prognostic markers of treatment outcome in patients with IC receiving primarily echinocandin therapy [16]; as well as serve as a guide to safely discontinue empiric antifungal treatment in culture-negative ICs. As most of our study patients stayed in the ICU for less than a week, we were unfortunately unable to evaluate these hypotheses.

Although not statistically significant, the median BDG values for infected IC patients were higher than those for colonized and non-colonized patients. A similar pattern was reported by Tissot where the median BDG in the patients with proven IC (223 pg/ml) was significantly higher than the other groups, including those with suspected and treated IC [14]. As such, a markedly elevated single BDG is strongly indicative of proven IC and was even shown to be associated with poor clinical outcome [17]. In our study, 1 of the 2 patients with BDG >500 pg/μl died of IC.

The BDG, CI and CS appear largely similar in their sensitivities, specificities, PPVs and NPVs. When the ROCs of the three modalities were analyzed, BDG provided the highest AUC of 0.89. It may be that the use of serial BDG levels may further increase the specificity and AUC correspondingly for detection of IC.

The main limitation of this study was the small number of subjects with proven IC as mentioned earlier. Despite liberal inclusion criteria, we had a difficult time recruiting patients not already receiving systemic antifungal therapy by day 3 of ICU admission.

## Conclusion

Colonization with *Candida* species in surgical patients is common, and can result in positive BDG test. BDG, CS and CI demonstrated high sensitivities but poor specificities in this study population. Given their high NPV, their utilities may lie in excluding IC instead. In particular, the kinetics of BDG in serial readings may instead be used in antifungal stewardship programs to guide discontinuation of empiric (culture-negative) antifungal treatments Nevertheless, BDG performed the best (AUC of 0.89) when compared with CS and CI in our study. Our findings need further validation.
